# Adolescent human immunodeficiency virus self-management: Needs of adolescents in the Eastern Cape

**DOI:** 10.4102/phcfm.v13i1.2756

**Published:** 2021-02-18

**Authors:** Leone Adams, Talitha Crowley

**Affiliations:** 1 Department of Nursing and Midwifery, Faculty of Medicine and Health Sciences, Stellenbosch University, Cape Town, South Africa

**Keywords:** self-management, adolescents, HIV, adolescents living with HIV, self-management programme

## Abstract

**Background:**

Human immunodeficiency virus (HIV) is a chronic illness and adolescents living with HIV (ALHIV) need the support of the whole family to self-manage (handle, direct and control) their chronic illness. Little is known about self-management amongst ALHIV in the context of the Eastern Cape, South Africa.

**Aim:**

This study explored the self-management needs of ALHIV in the Nelson Mandela Bay area of the Eastern Cape to make recommendations that can be used in further research to develop a programme to support adolescents with self-management.

**Setting:**

The study was conducted at two primary healthcare clinics in the Nelson Mandela Bay area of the Eastern Cape.

**Methods:**

A qualitative descriptive design was applied. Thirteen adolescents between the age of 14 and 19 years were interviewed. The data were collected through individual interviews. Data analysis was done using the six steps described by Creswell.

**Results:**

Adolescents living with HIV have limited knowledge and understanding about HIV and sexual reproductive health. Some ALHIV lack self-regulation skills related to decisions about disclosure, managing stigma and emotions, taking treatment, effective communication and setting goals. Human immunodeficiency virus services were not adolescent-friendly, with long queues and no dedicated services for adolescents. Family and friends were a key self-management resource for ALHIV.

**Conclusion:**

Adolescents living with HIV have several self-management needs in the domains of knowledge and beliefs, self-regulation skills and abilities, and self-management resources. Healthcare workers should support adolescents and their caregivers to acquire self-management skills as this may lead to better treatment and health outcomes.

## Introduction and background

Human immunodeficiency virus (HIV) is a chronic condition, and there is an increased focus on supporting patients with self-management. Self-management can be described as the interaction of health behaviours and related processes that patients and families engage in to take care of a chronic condition.^1^ To self-manage means to handle, direct and control one’s chronic illness. It includes components such as knowledge and beliefs, self-regulation and social facilitation or the utilisation of resources.^2^

Adolescents living with HIV (ALHIV) need the support of the whole family to self-manage their chronic illness. When the individuals and their families acquire self-management skills, they become responsible for the management of their chronic condition, are able to control the illness and acquire healthy behaviours by purposefully engaging in the performance of learned behaviour.^2,3^

An increased number of children living with the HIV are progressing to adolescence and beyond because of increased access to antiretroviral treatment (ART).^4^ The World Health Organization (WHO) defines adolescents as individuals in the 10–19 years age group. Early adolescents are between 10 and 14 years old and middle to late adolescents are 15–19 years old.^5^ Globally, 1.7 million adolescents aged between 10 and 19 years were living with HIV in 2019.^6^ At the time of the study, 455 adolescents in the age group of 14–19 years were on ART in the Nelson Mandela Bay District (NMBD) of the Eastern Cape. An estimated 20% of adolescents discontinued their treatment regime in 2016–2017.^7^

A systematic review by Millard et al. found evidence that self-management programmes for people living with HIV (PLHIV) result in short-term improvements in knowledge, physical and psychosocial health, and behaviour.^8^ Targeted self-management interventions, informed by thorough needs assessments, could improve health-related outcomes for PLHIV.^8^ Another systematic review found that self-management interventions for young people that focused on improving adherence or dealing with the chronic condition are effective irrespective of the chronic condition involved.^9^ Bernardin et al. report in their scoping review of self-management interventions for PLHIV that self-management interventions need to address a range of needs and should be tailored to a specific group and context.^10^

Globally, ALHIV tend to have poor treatment outcomes compared to adults, and viral suppression rates are concerning.^11^ A study that was done in clinics in Gauteng and Mpumalanga, South Africa, found that ALHIV and young adults, aged between 15 and 24 years, receiving ART tended to be virologically unsuppressed, have high loss to follow-up rates and have high virological failure rates compared to adults.^12^ There is, therefore, a need to support adolescents with self-management.

Only one South African study has specifically explored self-management amongst ALHIV. This study was conducted amongst 13- to 18-year-old ALHIV in Cape Town and identified several aspects of self-management with which adolescents needed support. The self-management aspects identified were coping with disclosure and stigma, participating in healthcare decisions and community activities, communicating about sensitive issues such as missing a dose of ART and sexual behaviours, knowledge of their viral load and names of antiretroviral drugs and remembering to take treatment.^13^ Higher levels of self-management were associated with treatment adherence, viral suppression and better health-related quality of life.^14^

Little is known about self-management amongst ALHIV in the context of the Eastern Cape, South Africa. Therefore, the self-management needs of adolescents in the context of the Eastern Cape were explored to make recommendations that can be used in further research to develop a programme to support adolescents with self-management.

## Theoretical framework

**FIGURE 1:**
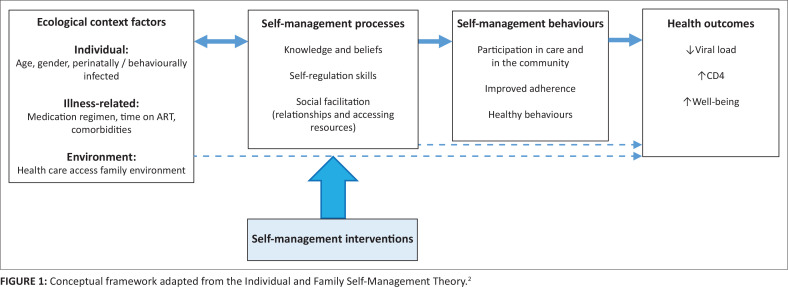
Conceptual framework adapted from the Individual and Family Self-Management Theory.^2^

The Individual and Family Self-Management Theory (IFSMT) is a middle-range theory that describes self-management in the socio-ecological context of the family and the individual, considering the physical and social environment as well as the characteristics that is unique to the family members. According to the IFSMT, self-management can be improved by facilitating knowledge and beliefs (such as information about one’s illness and self-efficacy), enhancing self-regulation skills (such as goal-setting and problem solving) and fostering social facilitation or self-management resources (such as social support).^2^ It is hypothesised that improvement in self-management processes leads to healthy behaviours and, subsequently, better health outcomes.^3^ The IFSMT provides a conceptual basis for the development of self-management interventions and was, therefore, used as an organising framework to identify the self-management needs of adolescents (see Figure 1).

There are other self-management theories and frameworks such as the Paediatric Self-Management Framework^1^ and the Self and Family Management Framework.^15^ However, the IFSMT was selected as it had been applied in the South African context to develop an instrument to measure adolescents’ HIV self-management.^16^

## Aim

The aim of the research was to explore the self-management needs of ALHIV in the Nelson Mandela Bay area of the Eastern Cape in order to make recommendations that can be used in further research to develop a programme to support adolescents with self-management. The specific objectives were to explore adolescents’ knowledge and beliefs, describe their self-regulation skills and identify the resources they use to facilitate self-management.

## Methods

### Research design

A qualitative exploratory-descriptive research design was used.

### Setting

Twenty-two clinics provide ART services to adolescents in the NMBD in the Eastern Cape. Primary healthcare clinics deliver a range of services such as chronic services, antenatal services, antiretroviral services and immunisation services. The prominent languages in the area are Afrikaans and Xhosa.

### Researcher’s position

The researcher is the first author and is a Professional Nurse working in the primary healthcare services of a clinic in the NMBD. The researcher has not worked at the two clinics where the study was conducted.

### Population and sampling

The researcher conducted the research at two clinics. The clinics are situated in the Northern areas of the NMBD and fall under the sub-district C area. The clinics were selected based on the number of adolescents accessing ART and accessibility to the researcher. The focus of this study was on older adolescents in the age group of 14–19 years (secondary school age). Older adolescents were chosen as opposed to younger adolescents because, based on their cognitive developmental stage, they are more capable of self-management skills such as self-monitoring, planning, goal setting and evaluation.^5^ It is also the age at which parents tend to transfer responsibility of care to the adolescent.^17^ At the time of the study, 30 ALHIV of this age group attended the one clinic and 35 ALHIV attended the second clinic.

In order to keep ALHIV’s personal details confidential, participants were recruited by asking the healthcare workers rendering ART services to refer potential participants. Inclusion criteria included the age group of 14–19 years and awareness of their HIV status. Healthcare workers confirmed whether the adolescent was aware of their HIV status to avoid accidental disclosure. The adolescents were recruited at the clinic, appointments scheduled and contact details obtained.

Purposive sampling was used to sample participants across different ages, languages and genders. Twenty adolescents were approached, two needed to be excluded because they were already 20 years old, and for five participants, parental consent could not be obtained or they did not return for interviews after several attempts to contact them. The sample size was determined by the emerging themes in the interviews and data saturation. Thirteen interviews were conducted, inclusive of the pilot interview.

### Data collection

Thirteen individual interviews were conducted by the researcher in a private place and at a time selected by the participants. A semi-structured interview guide was used. Interview questions started with building rapport, for example, ‘Tell me a bit about yourself’. This was followed by questions about their experiences at the clinics, when they had learned about their HIV-positive status and how they take care of themselves. Questions included, for example, ‘Tell me more about why you come to the clinic’; ‘Tell me more about your future plans’ and ‘Who supports you to take your treatment’? Probing questions were asked.

Interviews were recorded on an audio-recorder. Interviews were conducted in Afrikaans or English. The researcher is fluent in Afrikaans and English. Some of the participants were isiXhosa-speaking, but preferred to communicate in English, even though they were provided with the option of using an isiXhosa-speaking interpreter.

The researcher conducted one pilot interview with an adolescent living with HIV to test the interview guide questions. The data of the pilot interview were included in the main study. The researcher received training in conducting qualitative interviews prior to commencing the pilot interview and received continuous feedback regarding her interview technique from the study supervisor who listened to the audio recordings.

### Data analysis

Interviews were transcribed verbatim and checked by the first author. The six steps suggested by Creswell were used to analyse the data.^18^ This included organising and preparing data by checking the transcripts and labelling them, followed by reading the data and reflecting on the overall meaning. Both authors listened to the audio recordings and read the transcripts. The transcripts were coded by the first author, and the codes were checked by the second author by applying the eight steps described by Tesch (1992 in Creswell^18^). An iterative process occurred for each new transcript until a complete set of codes were developed. Both authors then re-checked the coding of the transcripts. Categories or sub-themes were generated by grouping related codes and themes by grouping sub-themes. After reaching agreement on the themes and sub-themes, the results were presented in a narrative by illustrating how the various themes are interconnected and interpreted by considering the context and identifying the lessons learned.

### Trustworthiness

To enhance credibility, the principles of prolonged engagement, reflexivity, recording of information and member checking were applied. The researcher has experience in providing services to ALHIV and thus had prior knowledge of their experiences. Field notes were taken whilst conducting the interviews, and a reflective diary was used to bracket the researcher’s personal feelings and experiences about the phenomenon as well as the data collection process. Although the researcher was a female and older than the adolescents, she did not experience difficulty in interviewing the adolescents. Boys and girls equally verbalised their experiences. However, older participants aged 17 to 19 years were more open and answered the questions in detail, whilst the younger participants aged 14 to 16 years had to be probed to answer questions.

Member checking was performed through probing to ensure that the true meaning of the participant’s experiences was understood. The researcher regularly spoke to the study supervisor and peers, who are experts, outside the study. Transferability was applied by providing thick descriptions of the participants and their context. An audit trail was kept of the data analysis as well as all communication between the supervisor and researcher. The supervisor checked the coding and themes.

### Ethical considerations

Ethical approval was obtained from the Health Research Ethics at the University of Stellenbosch, reference number#: S18/01/004. Permission was obtained from the Eastern Cape Department of Health, reference number, EC_201803_013.

During the recruitment of participants, gatekeepers such as caregivers were used to ensure that participants did not feel forced to participate. Informed consent forms were available in the home language of the potential participants. Adolescents under 18 years gave written informed assent and parental or guardian consent was obtained. Those who are 18 years and older provided their own consent. No participant needed referral because of becoming distressed during the interviews, and travel costs were reimbursed if incurred. To ensure confidentiality, no personal identifying information was recorded or transcribed.

## Results

### Participant characteristics

Two participants were behaviourally infected (acquired HIV through sexual transmission) with HIV, and 11 participants were perinatally infected (acquired HIV through mother-to-child transmission). Four participants were 14 years old, two participants were 16 years old, one participant was 17 years old, two participants were 18 years old and four participants were 19 years old.

### Themes

The themes and sub-themes are presented in Table 1.

**TABLE 1:** Themes and sub-themes.

Themes	Sub-themes
Knowledge and beliefs	Knowledge of HIV and sexual reproductive health Sources of information Reaction to living with HIV Self-esteem
Self-regulation skills	Decisions about disclosure Managing stigma Taking treatment Managing emotions Communication Setting goals
Self-management resources	Health facilities not adolescent-friendly Relationships with healthcare workers Support from family and friends

HIV, human immunodeficiency virus.

Three main themes were identified that encompassed the self-management processes as described in the IFSMT.

#### Knowledge and beliefs

Knowledge and beliefs include views and ideas about one’s illness, the future and confidence to self-manage.^2^ This study is concerned with what the participants knew and understood about HIV and sexual reproductive health as well as their feelings about living with HIV.

**Knowledge of human immunodeficiency virus and sexual reproductive health:** The participants lacked knowledge about HIV and sexual reproductive health. This included knowledge of how they acquired HIV:

‘I don’t know anything about HIV, I have to find out for myself.’ (Participant 13, 19-year-old female)

‘No, I don’t know how I got HIV.’ (Participant 10, 14-year-old male)

Certain adolescents demonstrated an understanding of safe sexual practices such as how to protect their partner from acquiring HIV. However, some participants, who were already sexually active, mentioned that they were pressurised by their boyfriends/girlfriends to have sex without condoms or found it challenging to negotiate condom use because of non-disclosure:

‘You must use a condom when you sleeping with a boy.’ (Participant 7, 14-year-old female)

‘I feel uncomfortable around my boyfriend, and he always ask why don’t we do it without a condom because we are first lovers, and I always have excuses.’ (Participant 11, 19-year-old female).

**Sources of information:** Several participants verbalised that they are scared or uncomfortable to talk to caregivers or guardians about HIV, sex or matters regarding life. They, therefore, accessed information about HIV by using the internet on their mobile phones or at home. A few participants mentioned that they received information from a family member or a close friend, and others mentioned getting information from the teachers or library at school:

‘I go to Google, mostly I used Google most of the time, but I also go to the library sometimes to study things that I found in Google to study in depth.’ (Participant 4, 18-year-old male)

‘I get information from the teachers at my school.’ (Participant 7, 14-year-old female)

The adolescents preferred to obtain information in a way with which they were comfortable. They were more comfortable searching for information on the internet than asking healthcare workers or caregivers.

**Reaction to living with human immunodeficiency virus:** The participants verbalised a range of reactions about living with HIV. These ranged from negative to positive feelings or a combination of both. Some of the negative feelings, such as anger and sadness, related to how they acquired HIV or the way in which the diagnosis was disclosed to them, for example, if their diagnosis was not communicated openly and honestly:

‘I felt so hurt because I didn’t know.’ (Participant 7, 14-year-old female)

‘I felt very hateful at first.’ (Participant 6, 19-year-old female)

Positive feelings, such as hope, emanated from their acceptance of the illness. One participant found hope in believing that scientists are still researching for an HIV cure. Other participants viewed their illness as something they have no agency to change:

‘I feel like I have it, there’s nothing I can do about it.’ (Participant 11, 19-year-old female)

**Self-esteem:** Self-esteem relates to the participants’ identity and concerns how the participants view themselves. Several participants were of the viewpoint that they can reach their potential in life despite living with HIV. The secrecy of their illness (other people being unaware that they are living with HIV) contributed to their good self-esteem as it gave them confidence that people will not perceive them as being different:

‘I don’t really look at myself as different.’ (Participant 2, 18-year-old male)

‘I see myself as the same person I was 5 years back before I discovered.’ (Participant 6, 19-year-old female)

#### Self-regulation skills

In this study, we identified self-regulation skills and abilities such as decisions about disclosure, managing stigma, taking treatment, managing emotions, communicating and setting goals as key self-regulation skills.

**Decisions about disclosure:** One of the skills an adolescent needs is the ability to know to whom and when to disclose their HIV status. One’s HIV status was viewed as being a personal issue and handled with extreme caution. It was apparent that the HIV status of the participants was not shared outside the family:

‘About first I was very cautious, I was small when she told me and said I shouldn’t tell anyone and so at that time all I felt was that I should not tell anyone because it is no one’s business. That is all I felt that I have to keep it secret. Its mine no-one else’s and I never felt the need to tell anyone.’ (Participant 4, 18-year-old male)

The participants made decisions about disclosure. In the context of relationships, they wanted to confirm trust and their long-term intentions for the relationship before taking the steps to disclose:

‘I will hide it for a bit until I trust the person then I will tell him or her that I am HIV positive.’ (Participant 11, 19-year-old female)

In other instances, disclosure was unplanned, but related to obtaining information, resources and support from others such as teachers:

‘I asked, ‘Teacher is there no one staying with you with HIV’? Then she say no. I ask about HIV and AIDS what it is about. Then she told me. She asked who have HIV, and I say me.’ (Participant 7, 14-year-old female)

**Managing stigma:** One of the more difficult self-management tasks is dealing or coping with stigma. Participants verbalised that stigma persists in communities:

‘Yes, they talk a lot, especially about my boyfriend, the one that gave me the virus, people talk about him, there were times when people used to ask me if I am not scared to be with him. I felt very bad.’ (Participant 6, 19-year-old female)

‘It will hurt if other people know, they will joke about it.’ (Participant 10, 14-year-old male)

The participants managed stigma differently. Some chose to ignore negative comments or interactions, whilst others found comfort and security in the fact that their HIV-positive status is confidential:

‘I don’t worry at all, because they don’t know that I have it.’ (Participant 12, 17-year-old female)

**Taking treatment:** Taking ART is an important part in managing the HIV infection. Treatment adherence leads to good health and control of the illness. Managing treatment included taking treatment daily and integrating it with other routines and activities.

‘I have a phone, then I look at the phone what is the time, when I see it is 12 o’clock then I go fetch water and drink it.’ (Participant 7, 14-year-old female)

To prevent accidental disclosure when taking treatment when the participants are with friends, they derived ways of taking their treatment to preserve their secret. This included taking treatment before going to their friends or excusing themselves from the group:

‘Ya, this one that was last year, it was in Grahamstown then there were lots of us, I made sure they are not seeing me going to the toilet to drink them and come back.’ (Participant 12, 17-year-old female)

**Managing emotions:** Participants had to manage emotions emanating from the lived realities of disclosure, HIV-related stigma and taking treatment daily. Emotional management varied from person to person. Avoidance and suppression of emotions were often used:

‘I don’t really talk, I feel that because it’s me, that’s the type of person I am.’ (Participant 2, 18-year-old male)

Participants demonstrated resilience by viewing HIV as only one aspect of their lives, thereby preventing negative emotions from taking over. They found comfort in talking to family or friends or their religion when they experienced feelings of sadness and needed encouragement:

‘People used to think I am arrogant. I never felt that people looking at me different, because I was never that kid that sits in the corner feel sorry for themselves. I don’t allow myself to do that.’ (Participant 4, 18-year-old male)

**Communication:** Young people with a chronic illness must communicate with caregivers, peers and healthcare workers. Communication between some participants, family and guardians and healthcare workers was challenging because of various reasons such as the generational gap and emotional discomfort to speak about HIV and related matters:

‘But my aunt is a person that is not easy to speak to, because she have a soft heart. So she just cried and I can’t stand to see her crying.’ (Participant 11, 19-year-old female)

Communicating with their guardian/parent about sex could imply that they are engaging in sexual activities, discouraging adolescents from engaging in such conversations:

‘No she is going to think that I also do it [*have sex*].’ (Participant 8, 16-year-old female)

Adolescents were demotivated to engage with healthcare workers because of the attitude of certain healthcare workers, a large number of patients at the clinics and long waiting times. They only engaged in a conversation if they felt it necessary:

‘It depends on the nurse that is consulting me. Sometimes I don’t have any questions. I read a lot about this already, so I only have a few questions on my mind that I will ask.’ (Participant 4, 18-year-old male)

**Setting goals:** Setting goals is an important component of self-management. The participants verbalised several goals. These were focused on obtaining an education, pursuing a specific career and having families of their own:

‘After school I want to go to Wits University to study social work.’ (Participant 12, 17-year-old female)

The majority of these goals were long-term goals, and several of the adolescents did not have a specific plan in place to attain these. They also did not have specific goals related to their own health and well-being.

#### Self-management resources

The resources that the participants used for social facilitation included health facilities, healthcare workers and family and friends.

**Health facilities not adolescent-friendly:** The health facilities did not provide a conducive environment for the participants to attend because of long queues and the lack of dedicated adolescent services. The participants expressed the need for a designated area for adolescents. Collecting treatment interrupted their school routine and led to lost school time:

‘When my mom is unavailable to come I come myself. I just find that the queues are really annoying.’ (Participant 4, 18-year-old male)

‘The lines are long, can’t they organise a place where we can get our treatment. You lose a whole day from school then you have to make up the next day.’ (Participant 6, 19-year-old female)

**Relationships with healthcare workers:** Adolescents’ experiences of healthcare workers varied from experiencing them as supportive to reporting that they provide limited information and support. Adolescents did not know what to expect from their interaction with healthcare workers and did not think that it was important for healthcare workers to engage with them. Interactions were focused on obtaining blood samples, giving blood results and dispensing treatment:

‘They just tell me about my viral load that’s it, that it is high.’ (Participant 11, 19-year-old female)

**Support from family and friends:** Having support and encouragement from friends and family allowed adolescents to continue taking their treatment, even at times when they become discouraged:

‘My friend is my confidante.’ (Participant 11, 19-year-old female)

‘We are a close-knit family that support each other through everything.’ (Participant 4, 18-year-old male)

One of the key support roles of the family is reminding the adolescent to take treatment. It appeared that if the participants received support from their family in taking their treatment, they were more conscientious in doing so. Some family members come to the facilities to collect the treatment for the participants:

‘My granny reminds me to take the treatment and she come to the clinic to get the treatment.’ (Participant 9, 14-year-old female)

## Discussion

The study identified a range of self-management needs in the domains of knowledge and beliefs, self-regulation skills and self-management resources.

### Knowledge and beliefs

Knowledge and beliefs influence behaviour, specifically self-efficacy, outcome expectancy and the integration of goals.^2^ Some of the participants demonstrated limited HIV and sexual reproductive health knowledge, even though they regularly access healthcare services. This is similar to a study conducted in the United States amongst youths (13–21 years old) that found ALHIV had limited understanding about their viral load and CD4 count.^19^ Disease-specific knowledge could assist adolescents to better monitor their illness. The findings emphasise that ALHIV need continuous counselling on HIV, sexual matters, family planning and treatment for sexually transmitted infections (STI) services.^20^

The internet (on their mobile phones) was identified as a frequently used source of information. The South African National Youth Health Policy recommends the use of technology in communicating with adolescents.^21^ Technology that particularly appeals to adolescents includes smartphones, apps, social and sexual networking services and games. These technologies are appropriate in the context of HIV because of the focus on anonymity, social support, real time assessment and feedback.^22^

The participants in the present study generally experienced positive feelings about living with HIV and had a good self-esteem. Good self-esteem was related to the perception that one will not be viewed as being different as long as your HIV-positive status is a secret. One of the reasons for a good self-esteem despite having a highly stigmatised illness may be good social support (emotional, instrumental or informational). Through social support from others, individuals can receive positive appraisal and manage negative feedback, which assures a positive evaluation of the self.^23^ The findings of this study resonate with a study conducted in Canada that found that adolescents saw themselves as being healthy human beings and that they had positive views of themselves and the future. The authors attributed this to access to ART, few side effects or problems with medication and medical and social support from a young age.^24^

### Self-regulation

In order to encourage ALHIV to self-manage their illness, they have to be taught skills like self-monitoring, planning, goal-setting and evaluation.^5^ In the present study, self-regulation abilities included decisions about disclosure, taking treatment, managing stigma and emotions, communication and setting goals.

Human immunodeficiency virus disclosure was limited to the immediate family. Similar disclosure practices have been reported in Zambia and South Africa^20,25^ and may be because of anticipating HIV-related stigma. Learning how to manage HIV-related stigma is crucial as various forms of stigma in families, communities and healthcare settings have been identified in our study and in other studies conducted amongst ALHIV in Africa.^26,27,28^

Integrating treatment into daily routines is an important self-regulation skill, and participants were able to set reminders and developed strategies for taking their treatment when they were with friends to prevent accidental disclosure. A study that was done in Zambia reported that the youth often delayed taking their medication for several hours when playing with friends or did not use ART when travelling away from home to prevent unintended disclosure. Like the present study, the majority of adolescents had to be reminded by their families to take their treatment.^25^

Managing emotions can be complex for an adolescent. Strategies to manage emotions ranged from avoidance to engaging the support of others, specifically family members. Adolescents living with HIV are at greater risk of internalising symptoms if they experience HIV-related stigma and tend to use avoidance to manage negative emotions.^26^ In a study done in Kampala, it was found that adolescents found comfort in the knowledge that they were not the only persons living with HIV.^29^

In general, the participants found it challenging to communicate with parents or guardians, and especially grandparents, about their feelings, HIV and sex. The poor communication between the participants and caregivers or parents also led to ineffective disclosure practices such as late disclosure. Adolescents appreciate open and truthful communication from parents and caregivers.^30^ Long queues or not feeling comfortable enough to ask questions prevented communication with healthcare workers. Similarly, in a study done in the United Kingdom, Ireland, Uganda and the United States, the majority of adolescents reported not feeling comfortable to ask healthcare workers questions and relied on fragmented information from the internet to make sense of their situations.^31^

The participants in the study had a positive outlook that included career and family goals. However, it was mostly the older participants who had thought of how they might go about achieving their goals. Programmes that assist adolescents with self-management should include career planning.^5^ Adolescents also need to set health goals and healthcare providers must engage the youth, so they understand the current status of their illness.^19^ Adolescents need to participate in their own care by, for example, drawing up action plans that focus on illness needs, accessing resources, dealing with symptoms and asking for support when needed.^32^

### Self-management resources

People are more likely to engage in recommended health behaviours if they experience social facilitation. Social facilitation includes social influence, social support and collaboration between the individual, family and healthcare providers.^5^

From the perspective of the participants, the health facilities and services are not conducive to keeping appointments because of the lack of dedicated adolescent services, long waiting times and clashes with school schedules. In a study that was done in the Eastern Cape, it was found that the waiting times and adolescents’ experiences at the clinic (i.e. confidentiality concerns) had an impact on retention in care.^33^

The participants experienced limited communication and support from healthcare workers. A study that was done in the Eastern Cape of South Africa found that if adolescents perceived healthcare providers as being kind and having time for them, the odds of retaining them in care increased 2.5 times.^33^

Several participants had support from their family. Family members supported adolescents by reminding them to take their treatment or went to the clinic to collect treatment on their behalf. Living with an adult who provides daily support for HIV treatment, and who is also living with HIV, helped to normalise the lives of ALHIV in Canada.^24^ In a study that was done in Zambia, it was found that in addition to verbal reminders, many participants’ families provided them with emotional support.^20^ Being accompanied to the clinic was associated with retention in care in a study in the Eastern Cape.^33^

### Recommendations

Recommendations for self-management were identified based on the study findings and according to the self-management components (Table 2).

**TABLE 2:** Summary of self-management recommendations based on the identified needs.

Self-management component	Self-management needs	Recommendations
Knowledge and beliefs	Lack of HIV and sexual reproductive health knowledge; Acceptance and self-esteem	1. Implement strategies to improve HIV and sexual health knowledge, positive feelings and self-esteem. 2. Use information-communications-technology (ICT) platforms.
Self-regulation skills	Disclosure decisions; Stigma and emotion management; Taking treatment; Communication challenges; Goal setting	3. Implement strategies focused on improving a range of self-regulation skills focused on the identified needs such as integrating the 5 A’s model (Assess, Advise, Agree, Assist, Arrange) for providing self-management support^5^ into routine consultations.
Self-management resources	Lack of adolescent-friendly services; Relationships with healthcare workers; Family and peer support	4. Provide adolescent-friendly services, for example, dedicated service areas and times. 5. Train healthcare providers to work with adolescents, including how to provide self-management support using the 5 A’s approach.^5^ 6. Increase family and peer involvement.

HIV, human immunodeficiency virus.

### Limitations

A limitation was that some of the adolescents, who needed the consent of their guardians or parents, did not return for the interview with the consent forms or did not return at all and, consequently, could not participate. There were adolescents who did not know their HIV status and therefore had to be excluded from participating and were not referred to the researcher. Although some of the participants were isiXhosa-speaking, they indicated that they were comfortable with communicating in English. This could have led to possible bias or lack of in-depth understanding on the part of the researcher. However, the researcher continued with interviews until data saturation was reached.

## Conclusion

A range of self-management needs of ALHIV were identified in the domains of knowledge and beliefs, self-regulation skills and self-management resources. To address the self-management needs, self-management programmes for adolescents should be comprehensive and focus on strategies to enhance HIV and sexual reproductive health knowledge, acceptance of the disease and positive self-esteem. Information should preferably be communicated via information-communications-technology (ICT) platforms. It is important to implement strategies focused on improving self-regulation skills, as well as providing adolescent-friendly services. Moreover, it is necessary to train healthcare providers to work with adolescents and provide self-management support, whilst encouraging family and peer involvement.
